# Designer *π*-magnetism in magnetic graphene nanostructures: advances and future perspectives

**DOI:** 10.1093/nsr/nwag157

**Published:** 2026-03-11

**Authors:** Xinnan Peng, Wenlong E, Yu Teng, Haoyu Zhang, En Li, Yu Wang, Lulu Wang, Shaotang Song, Jiong Lu

**Affiliations:** Department of Chemistry, National University of Singapore, Singapore 117543, Singapore; Department of Chemistry, National University of Singapore, Singapore 117543, Singapore; Department of Chemistry, National University of Singapore, Singapore 117543, Singapore; Department of Chemistry, National University of Singapore, Singapore 117543, Singapore; Department of Chemistry, National University of Singapore, Singapore 117543, Singapore; Department of Chemistry, National University of Singapore, Singapore 117543, Singapore; Institute for Functional Intelligent Materials, National University of Singapore, Singapore 117544, Singapore; Department of Chemistry, National University of Singapore, Singapore 117543, Singapore; Interdisciplinary Materials Research Center, School of Materials Science and Engineering, Tongji University, Shanghai 201804, China; Department of Chemistry, National University of Singapore, Singapore 117543, Singapore; Institute for Functional Intelligent Materials, National University of Singapore, Singapore 117544, Singapore

**Keywords:** magnetic graphene nanostructures, molecular magnet, on-surface synthesis, scanning probe microscopy

## Abstract

Magnetic graphene nanostructures (MGNs) represent a rapidly advancing frontier in molecular quantum materials, distinguished by *π*-magnetism that arises from the topological design of their *π*-electron networks. The *π*-magnetism and correlated quantum phases in these systems can be precisely engineered through deliberate control of the molecular topology, sublattice symmetry and electron correlation, transforming low-dimensional carbon-based architectures into versatile model platforms for exploring exotic quantum phenomena. Recent advances in on-surface synthesis have further propelled this field by enabling the atomically precise fabrication of MGNs and fine control over their electronic and magnetic properties. Complementing these synthetic advances, progress in low-temperature scanning probe microscopy now affords unprecedented capabilities to characterize individual *π*-spins, exchange coupling and correlated ground states at the single-molecule level. Together, these developments have established a robust foundation for exploring long-lived spin coherence, tunable quantum entanglement and spin-based logic operations in carbon-based systems. This review highlights recent conceptual and methodological advances, emphasizing how rational molecular design, atomically precise synthesis and state-of-the-art characterization techniques collectively advance the understanding and realization of *π*-magnetism in MGNs. Remaining challenges, including stabilizing chemically reactive open-shell structures, mitigating substrate-induced hybridization and integrating molecular magnets into functional device architectures, are also discussed. Continued progress in this field will reshape our perspective on designing novel forms of magnetism in conjugated organic materials and open up new pathways toward scalable molecular spintronics and quantum technologies.

## Teaser

This review illustrates how designed graphene nanostructures can function as tunable quantum magnets, highlighting their atomically precise fabrication and characterization methods that open new opportunities for future quantum devices.

## INTRODUCTION

**Figure 1.**
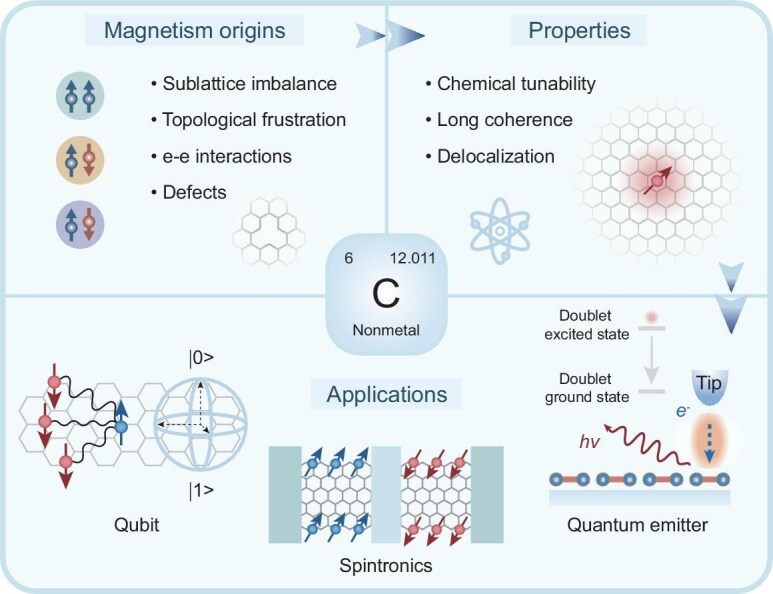
Schematic illustration of the origins, fundamental properties and potential applications of *π*-magnetism in magnetic graphene nanostructures (MGNs).

Magnetic graphene nanostructures (MGNs) are emerging as promising building blocks for designing unconventional *π*-magnetism and strongly correlated quantum phases for next-generation quantum technologies [1,2] (Fig. 1). Unlike conventional magnetic materials based on the *d*- or *f*-electrons of transition metals or lanthanides, magnetism in these carbon-based systems arises from unpaired *p_z_* electrons, typically found at zigzag edge segments or defect sites or heteroatom sites that disrupt the otherwise paired *π*-electron networks. Due to the absence of heavy atoms, the resulting *π*-magnetism in MGNs, characterized by intrinsically weak spin–orbit coupling, sparse nuclear spin bath and extended spin correlation lengths, offers a versatile platform for realizing robust molecular qubits [3,4], coherent spin chains [5,6] and high-performance spin filters [7].

The rapid development of MGNs has significantly reshaped our understanding of *π*-magnetism in conjugated carbon networks. Different theoretical models, such as Lieb’s theorem [8,9] and graph theory [10], provide powerful tools for predicting ground-state spin multiplicity and exchange coupling strengths. These principles often act cooperatively, giving rise to a broad spectrum of magnetic behaviors, from ferromagnetic and antiferromagnetic order to bistable and even quantum-entangled ground states [11–14]. Consequently, the magnetic properties of MGNs are inherently governed by a delicate interplay between geometrical topology [15,16], electron correlations [17,18] and structural architecture [19,20], enabling the precise control of spin states through rational molecular design.

Despite considerable theoretical progress, the realization of MGNs with atomic precision remains experimentally challenging. Conventional solution-phase synthesis often suffers from poor solubility, radical instability and limited access to single-molecule magnetic characterization [15]. In contrast, on-surface synthesis (OSS) has emerged, over the past decades, as a powerful route to fabricate atomically precise graphene nanostructures. In this approach, rationally designed precursor molecules, synthesized in solution, are deposited onto atomically clean metal surfaces under ultrahigh vacuum conditions. Subsequent thermal annealing, electron injection, ultraviolet irradiation or tip-induced manipulation then drives covalent transformations that yield the desired nanostructures with atomic precision [21–27]. Additionally, high-resolution scanning probe techniques such as scanning tunneling microscopy (STM) and non-contact atomic force microscopy (nc-AFM) enable direct visualization of molecular structures and detailed spectroscopic studies of electronic and magnetic fingerprints at the single-molecule level [12,13,28]. Recent breakthroughs have demonstrated that magnetism can be deliberately imprinted into these nanostructures, exemplified by triangulenes [11,29–32] and Clar’s goblet [12], stimulating extensive exploration of diverse MGNs, such as open-shell (OS) diradicals and polyradicals with well-defined spin multiplicities. These advances bridge theoretical predictions with experimental realization and establish MGNs as high-precision platforms for carbon-based quantum nanoscience and device applications [1–7].

To study fundamental spin physics and exotic quantum phases, it is essential to probe magnetic states at the atomic scale, necessitating experimental techniques with both atomic-structure resolution and high spin sensitivity. Advanced methods such as STM and scanning tunneling spectroscopy (STS) have been instrumental in identifying local magnetic moments and spin excitations in OS molecular systems. However, the spin states can be severely obscured or perturbed by factors such as substrate-induced hybridization, charge transfer and the intrinsic spectral resolution limitations of STS (i.e. linewidth and broadening), particularly in systems featuring quenched-edge magnetism or small spin-splitting energies. These limitations underscore the need for new spectroscopic approaches, improved substrate-decoupling strategies and integrated multimodal characterization capable of capturing the full spin physics of quantum magnetic systems.

Collectively, advances in molecular design, precise synthesis and characterization are redefining the landscape of MGNs. Their scalability, together with tunable chemical structures and spin states, points toward a new era of encoded quantum functionality. Nevertheless, significant challenges remain, especially in achieving precision magnetic characterization and stabilizing OS systems under practical conditions. Addressing these challenges through advances in synthesis, substrate engineering and high-resolution spin spectroscopy will be crucial for transforming proof-of-concept magnetic nanostructures into future quantum devices.

Although some reviews exist on the OSS and characterizations of MGNs using advanced scanning probe microscopy [15,16], this field is rapidly evolving. Significant recent advances, particularly concerning strongly correlated polyradicals and novel magnetism-probing techniques, necessitate an updated summary. This review provides a comprehensive overview of recent developments, highlighting both conceptual progress and experimental frontiers. The discussion is organized into four sections: (i) ‘Origins of π-magnetism and design principles’, examining how sublattice symmetry, *π*-electron topology and electron correlation govern ground-state spin multiplicities and exchange coupling; (ii) ‘Tuning spin states via molecular topology and structural defects’, covering how topological design, heteroatom substitution and non-hexagonal ring incorporation expand the scope of *π*-magnetism engineering; (iii) ‘Polyradical nanographenes with strong spin entanglement’, focusing on correlated multi-spin systems; and (iv) ‘Advances in techniques for probing nanoscale magnetism’, evaluating the latest characterization methods designed to overcome the limitations of conventional approaches.

## ORIGINS OF *Π*-MAGNETISM AND DESIGN PRINCIPLES

*π*-Magnetism in graphene nanostructures originates from sublattice imbalance, topological frustration and electron–electron (e–e) interactions, which prevent the complete pairing of *π*-electrons. Breakthroughs in solution-phase synthesis and on-surface fabrication have yielded prototypical systems such as triangulenes and Clar’s goblet [11,12,29–33], enabling direct probing of their ground-state multiplicities, exchange coupling strengths and spin excitations via low-temperature STM/AFM, STS and inelastic electron tunneling spectroscopy (IETS). Collectively, these studies establish a chemically programmable relationship between structure and spin properties. Building on this foundation, the following sections examine the mechanistic origins of *π*-magnetism in nanographenes and outline the general design principles for constructing tunable *π*-spin systems.

### Sublattice imbalance

**Figure 2.**
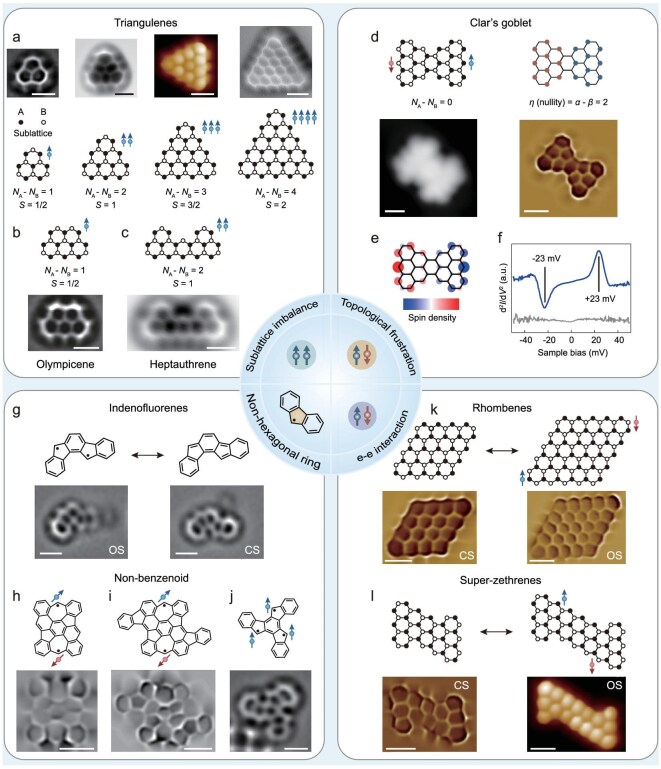
Origins of *π*-magnetism. (a) Chemical structures of [2], [3], [4] and [5]triangulene and their corresponding nc-AFM images. Reproduced with permission from Refs [11,29,30,32]. (b, c) Chemical structures of (b) olympicene and (c) heptauthrene, together with their corresponding nc-AFM images. Reproduced with permission from Refs [34,35]. (d) Chemical structure (top left) and schematic illustration of the ‘nullity’ (top right) of Clar’s goblet, along with its corresponding STM/nc-AFM images (bottom). The colored sites represent a maximum set of non-adjacent sites. (e) Mean-field Hubbard-calculated spin-density distribution. (f) d^2^*I*/d*V*^2^ spectra acquired near the Fermi level, revealing inelastic spin excitation at ±23 mV. Reproduced with permission from Ref. [12]. (g) Chemical structures and corresponding nc-AFM images of OS (left) and CS (right) indenofluorenes, the site-dependent configuration of which can be controlled via tip manipulation. Reproduced with permission from Ref. [53]. (h) Chemical structure of an azulene-embedded non-benzenoid nanographene and its corresponding nc-AFM image. Reproduced with permission from Ref. [50]. (i) Chemical structure of an extended nanographene derived from (h) and its corresponding nc-AFM image. Reproduced with permission from Ref. [51]. (j) Chemical structure of truxene-5,10,15-triyl and its corresponding nc-AFM image. Reproduced with permission from Ref. [19]. (k) Chemical structures of CS [4]rhombene (left) and OS open-shell [5]rhombene (right), along with their corresponding nc-AFM images. Reproduced with permission from Ref. [17]. (l) Chemical structures of closed-shell super-heptazethrene (CS, left) and open-shell super-nonazethrene (OS, right), along with their corresponding nc-AFM images. Reproduced with permission from Refs [44,45]. *N*_A_ and *N*_B_ denote the number of sublattices A and B. All scale bars: 0.5 nm.

Graphene lattice consists of two interlaced hexagonal sublattices, A and B, with chemical bonds forming exclusively between carbon sites belonging to opposite sublattices, giving rise to a bipartite structure (Fig. 2a). In the half-filled bipartite lattice systems, when the numbers of carbon atoms on the two sublattices differ (*N*_A_ ≠ *N*_B_), the imbalance forces some *p_z_* orbitals of the major sublattice to be singly occupied, producing *π*-radicals that each carry spin *S* = 1/2 and giving rise to intrinsic magnetism. This topology–spin relationship is formalized in Lieb’s theorem [8], an extension of Ovchinnikov’s rule [9], which predicts the total spin as *S* = ½|*N*_A_ − *N*_B_| (the factor ½ reflects the spin quantum number of the electron). Guided by this principle, researchers have designed molecular systems with built-in sublattice imbalance, enabling the controlled synthesis of *π*-magnetic nanographenes on surfaces.

The triangulene family provides a canonical demonstration of sublattice imbalance-driven magnetism (Fig. 2a). In these triangular *π*-systems with zigzag edges, the number of spins scales with molecular size (*n*), directly reflecting the sublattice imbalance. For instance, [3]triangulene (*n* = 3) contains 12 atoms on sublattice A and 10 on sublattice B, yielding a predicted *S* = 1 ground state with ferromagnetically aligned *π*-radicals, consistently with the theory [11]. Recent advances in OSS have enabled the fabrication of a series of triangulene homologues, including [2], [3], [4], [5] and [7]triangulene, allowing the systematic tuning of magnetic ground states by molecular size [11,29–32]. Beyond triangulenes, various sublattice-imbalanced nanographenes have been synthesized on surfaces, hosting intrinsic *π*-magnetism. Representative cases include olympicene with *S* = 1/2 ground state (Fig. 2b) [34] and heptauthrene with *S* = 1 ground state (Fig. 2c) arising from two corner-localized radicals on opposite edges [35]. Together, these systems illustrate the general scaling rule *S* = ½|*N*_A_ − *N*_B_| and highlight how precursor design and substrate coupling govern the manifestation of intrinsic *π*-magnetism.

### Topological frustration

Topological frustration can arise in *π*-conjugated networks that are sublattice-balanced yet still unable to support a closed-shell (CS) Kekulé resonance without leaving unpaired electrons. In such bipartite *π*-networks, the nullity (*η*), which counts the number of non-bonding molecular orbitals or zero-energy modes (ZMs), serves as a quantitative descriptor of electronic frustration (Fig. 2d). According to the graph theory introduced by Fajtlowicz and co-workers [36], $\eta = 2\alpha - N = \ N - 2\mu $, where *N* is the number of carbon sites, *α* is the size of a maximum independent set (the largest set of non-adjacent vertices) and *μ* is the size of a maximum matching (the largest number of disjoint C–C pairs). Physically, *η* represents the number of ZMs in the nearest-neighbor tight-binding picture and therefore the minimum number of unpaired *π*-electrons in any resonance structure. For systems with *N*_A_ − *N*_B_ = 0 but *η* > 0, Lieb’s theorem predicts a net spin of *S* = 0. The ground state is typically an OS singlet composed of antiferromagnetically coupled spin centers (Fig. 2e). The magnitude of the corresponding exchange coupling (*J*) depends sensitively on the symmetry and spatial overlap of the ZMs [10]. Thus, *η* serves as a concise design metric, distinguishing CS (*η* = 0) from frustrated OS (*η* > 0) nanographenes.

A classic realization of topological frustration is Clar’s goblet (Fig. 2d)—a bowtie-shaped nanographene (C₃₈H₁₈) first proposed by Clar in 1972. In this structure, the *π*-electron network cannot accommodate a CS Kekulé structure without leaving unpaired electrons, yielding a magnetically nontrivial ground state. The first single-molecule synthesis of this structure was reported by Mishra *et al.*, who performed high-resolution STS to characterize the spin properties of individual molecules adsorbed on Au(111) [12]. Their measurements revealed a robust antiferromagnetic ground state with an exchange coupling of 23 meV (Fig. 2f)—well above the Landauer bound for minimal energy dissipation at room temperature.

### Electron–electron interactions

Beyond topological frustration, coulombic e–e interactions constitute another fundamental mechanism driving spin polarization in carbon-based nanostructures [37,38]. In systems hosting ZMs, the splitting of spin-up and spin-down states in space (spin-symmetry breaking) minimizes the Coulomb repulsion. Even without ZMs, this spin-symmetry breaking still happens when the energy penalty for double occupancy exceeds that of forming spatially separated singly occupied orbitals [39]. The strength of this spin-symmetry breaking depends on both the orbital delocalization and the energetic proximity to the Fermi level [40]. Extended *π*-systems with small energy gaps tend to develop strong spin-symmetry breaking, whereas smaller systems tend to hybridize radical states into CS configurations [17,18]. These correlation effects highlight the widely observed size-dependent emergence of magnetism in nanographenes.

Recent experimental findings have established a correlation-driven, size-tunable route to *π*-magnetism, as exemplified by the emerging OS configurations in long acenes [41–43]. Notably, Fasel and co-workers demonstrated a size-dependent magnetic onset in rhombus-shaped zigzag nanographenes: [4]-rhombene remains CS, whereas [5]-rhombene exhibits an OS singlet ground state with a singlet–triplet gap of ∼102 meV (Fig. 2k), surpassing the Landauer limit [17]. Substrate effects further modulate magnetic robustness: charge transfer into singly occupied molecular orbitals quenches the spin on Ag(111), illustrating how electron correlation and substrate hybridization jointly determine magnetic behaviors. As a non-alternant counterpart, super-nonazethrene, the largest super-zethrene synthesized on Au(111), exhibits a frontier orbital gap of ∼1.0 eV and correlation-induced singlet–triplet excitations consistent with an OS singlet ground state (Fig. 2l) [44,45]. The extracted *J* of ∼51 meV and the gap reopening relative to smaller homologues highlight how correlation-driven spin splitting can promote the emergence of *π*-magnetism in extended nanographenes. Furthermore, approaching the CS/OS transition yields the strongest magnetic exchange coupling. Biswas *et al.* predicted this transition across three zigzag-rich nanographene families and subsequently synthesized the smallest OS members on Au(111) [18]. IETS revealed exceptionally large exchange couplings of ∼116, 183 and 190 meV, confirming that molecules positioned near the transition boundary host the most pronounced coupling strengths.

### Non-hexagonal rings

Beyond sublattice imbalance, topological frustration and e–e interaction, another topological route to *π*-magnetism arises from incorporating odd-membered rings, most notably pentagons and heptagons, into otherwise benzenoid frameworks. These non-hexagonal units disrupt the bipartite symmetry of the graphene lattice and frustrate local Kekulé pairing, thereby introducing topological defects that promote the emergence of unpaired *π*-electrons. Depending on the number and arrangement of these defects, the resulting ZM orbitals can host isolated *S* = 1/2 radical centers or couple to produce high-spin ground states (*S* ≥ 1). In graph-theory frameworks, these non-hexagonal defects modify the maximum matchings and typically increase the nullity (*η*), thereby generating ZMs that cannot all be spin-paired. Representative motifs include azulene (fused 5/7 pair) units embedded within nanographenes [46] or graphene nanoribbons [47], pentagon-mediated edge reconstructions and 5/7 dislocation structures arising from Stone–Wales defect chains [48,49].

Recent advances have enabled the OSS of magnetic nanographenes featuring such non-hexagonal motifs (Fig. 2h). Extended azulene-embedded non-benzenoid nanographenes containing two azulene units have been constructed through on-surface cyclodehydrogenation (Fig. 2i) [50,51]. Bond-resolved STM (BR–STM) imaging and STS measurements on Au(111) revealed an ultra-narrow frontier gap of ∼0.27 eV and near-unity biradical character (≈0.92), in quantitative agreement with multireference calculations. To access the higher-spin ground states in non-benzenoid systems, Gross, Wang and their co-workers employed the tip-induced dehydrogenation of truxene to generate the non-Kekulé triradical truxene-5,10,15-triyl, which maintains a quartet (*S* = 3/2) ground state on bilayer NaCl/Cu(111) (Fig. 2j) [19,52]. Constant-height imaging resolved the spin-split singly occupied molecular orbitals (SOMOs) and key reaction intermediates, including fluorenyl radical and indeno[1,2-a]fluorene, were directly observed along the reaction pathway. Extending this strategy from static magnetism to bistable magnetism, the same team generated unsubstituted indeno[1,2-a]fluorene through tip-induced C–H cleavage on bilayer NaCl/Au(111) (Fig. 2g) [53]. Remarkably, the neutral molecule switches reversibly between an OS *π*-diradical (predicted as triplet) and a CS para-quinoidal form. On lower-work-function substrates, it further exhibits neutral/anionic charge bistability, in which the two ground states interconvert simply by lateral displacement on the surface. These findings demonstrate how non-hexagonal rings enable spin polarization on specific sites, opening up opportunities for molecular spin logic and quantum information applications.

## TUNING SPIN STATES VIA MOLECULAR TOPOLOGY AND STRUCTURAL DEFECTS

The design and control of low-dimensional magnetic systems are central to unraveling strongly correlated physics and exploring exotic quantum phases. Beyond understanding the origins of magnetism, a key challenge lies in precisely controlling magnetic properties, which are governed by both molecular topology and structural defects. Relevant parameters include molecular size, edge configuration, the presence of nanopores, structural symmetry, as well as the incorporation of heteroatom dopants and topological defects [15,16,54]. Establishing how these factors dictate *π*-magnetism is therefore essential for the rational design and control of carbon-based magnetic nanostructures.

### Molecular topology engineering

**Figure 3.**
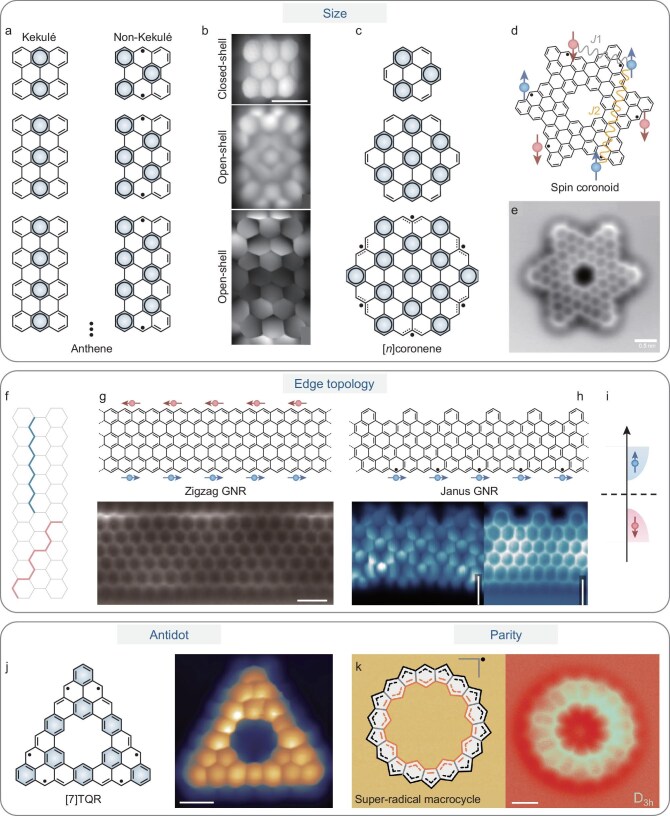
Tuning spin states via molecular topology engineering. (a) CS Kekulé (left) and OS non-Kekulé (right) structures of anthene derivatives with varying sizes. (b) Corresponding nc-AFM images of the anthene series. Reproduced with permission from Ref. [55]. (c) Hexagonal [*n*]coronenes featuring six zigzag edges, exhibiting size-dependent OS polyradical character. (d) Chemical structure of an OS coronoid with sketch of the nearest- and next-nearest-neighbor exchange interactions (*J*_1_ and *J*_2_, respectively). (e) Corresponding nc-AFM image. Reproduced with permission from Ref. [60]. (f) Schematic illustration of zigzag and armchair edge structures. (g) Chemical structure of a zigzag GNR (top) and its corresponding nc-AFM image (bottom). Reproduced with permission from Ref. [14]. (h) Chemical structure of a Janus GNR (JGNR) (top) and its corresponding BR–STM and nc-AFM images (bottom). Reproduced with permission from Ref. [69]. (i) Schematic diagram of the spin-polarized band structure near the fundamental bandgap of JGNR, exhibiting ferromagnetic order localized at a single zigzag edge. (j). Chemical structure of a [7]triangulene quantum ring ([7]TQR) and its corresponding BR–STM image. Reproduced with permission from Ref. [76]. (k) Chemical structure of a super radical macrocycle and its corresponding nc-AFM image. Reproduced with permission from Ref. [78]. All scale bars: 0.5 nm.

A widely adopted strategy for tuning the number of unpaired electrons and their interactions is to modify the molecular size. For instance, the number of ferromagnetically coupled spins in triangulenes increases linearly with their size (Fig. 2a), while, in rhombenes (Fig. 2k), the strength of the e–e interactions and the onset of additional spins exhibit a critical size dependence. Extending this concept, Cai and co-workers synthesized anthene homologues of different lengths on the Au(111) surface by using a co-deposition approach (Fig. 3a and b) [55]. The anthene family reveals a transition from CS configurations in smaller species to antiferromagnetic OS singlets in larger ones. This transition arises from the interplay between hybridization energy and Coulomb repulsion among valence electrons, both highly sensitive to molecular size and shape. The progressive narrowing of the frontier orbital energy gap with increasing length further elucidates the link between charge transfer and magnetic properties. The size-dependent modulation of magnetic properties in anthene nanostructures thus exemplifies how the controlling *π*-conjugated length governs their spin characteristics, complementing insights from rhombenes and super-zethrenes.

Similarly, hexagonal graphene nanostructures with six zigzag edges (denoted as [*n*]coronene, where *n* represents the number of carbon atoms per edge) have been theoretically predicted to adopt OS ground state beyond a critical size (Fig. 3c). For instance, [2]- and [3]coronene remain CS, whereas larger [4]coronene exhibits a multiradical OS character with six unpaired electrons [15,56,57]. Experimentally, the synthesis of large coronene homologues poses significant challenges due to their high chemical reactivity, necessitating a rational precursor design for controlled OSS. Recently, we demonstrated a surface-assisted synthetic route enabling ultrahigh yield fabrication (>98%) of circumcoronene and its superlattice on Cu(111) [58]. This success relies on a specially designed precursor comprising a coronene core decorated with six pairs of methyl groups on the peripheral benzene rings, effectively suppressing undesired side reactions. This strategy establishes a general framework for synthesizing large OS coronene derivatives through rational molecular design. The synthesis of coronoid, another family of large polycyclic systems, has long been considered formidable, particularly for OS variants in which the radical character emerges only beyond a critical molecular size [59]. Yu and co-workers recently realized such an open-shell coronoid hosting six unpaired electrons via OSS (Fig. 3d and e) [60]. This spin coronoid hosts collective magnetic states mediated by both nearest- and next-nearest-neighbor exchange interactions along conjugation pathways, as revealed by using IETS.

The size-dependent radical characteristics observed across triangulenes, rhombenes, anthenes, coronenes and coronoids can also be rationalized by using Clar’s sextet rule. This principle states that the most stable resonance structure of a polycyclic aromatic hydrocarbon requires a balance between the number of radical sites and the number of disjoint aromatic *π*-sextets (benzene-like rings containing six delocalized *π*-electrons). As nanographenes expand in size, more sextets can be formed with an increasing number of spin centers, enhancing resonance stabilization while simultaneously promoting the emergence of OS configurations once the fully sextet-stabilized limit is exceeded (Fig. 3a) [61].

In addition to molecular size, edge configuration serves as another critical parameter governing the magnetic properties of graphene nanostructures. Cutting graphene along different directions produces two primary edge configurations: armchair and zigzag edges (Fig. 3f). Among them, zigzag-edged structures are particularly intriguing as the nonuniform chemical environment of carbon atoms from sublattices A and B, making a natural imbalance that contributes to the magnetization [62]. Furthermore, zigzag-edged graphene nanoribbons (ZGNRs) are predicted to host metallic edge states due to the overlap of unsaturated *sp*^2^-orbitals, while most armchair-edged graphene nanoribbons remain semiconducting [63,64]. In line with these predictions, Fasel and co-workers achieved the first experimental synthesis of a ZGNR from a precisely engineered precursor that directed the formation of atomically defined zigzag edges (Fig. 3g) [14]. STM and nc-AFM unambiguously resolved the zigzag edge structure, while STS measurements revealed a splitting of the resonance peaks near the Fermi level upon NaCl-monolayer decoupling, which is attributed to the antiferromagnetic coupling between the highly localized edge states on opposite sides of the ribbon. This milestone provided the first direct confirmation of theoretically predicted magnetic edge states in ZGNRs.

Building upon the magnetic and topological properties of ZGNRs, recent efforts have focused on manipulating their *π*-electron topology to access new quantum phases. In symmetric ZGNRs, spin-polarized edge states on opposite sides typically couple antiferromagnetically. Suppressing this cross-edge interaction offers a route to realizing ferromagnetically ordered quantum spin chains [65], enabling the study of 1D quantum spin physics and quantum coherence [66,67]. Furthermore, achieving such carbon-based ferromagnetic transport channels is crucial for the ultimate miniaturization of graphene nanoribbon (GNR)-based quantum electronics [66,68]. In this context, our group recently reported a general approach for the design and fabrication of ferromagnetic GNRs in the form of Janus GNRs (JGNRs) featuring two distinct edge configurations (Fig. 3h) [69]. Guided by Lieb’s theorem and topological classification of the chiral phase index [8,70,71], we engineered two JGNRs by asymmetrically introducing a periodic array of benzene-ring defects along one zigzag edge while leaving the opposite edge intact. This symmetry breaking generates a sublattice imbalance within each unit cell, leading to spin polarization and stabilization of a ferromagnetic ground state (Fig. 3i). To realize this concept, three Z-shaped precursors were designed to yield one parent ZGNR and two JGNRs with optimized defect spacing, ensuring the complete quenching of magnetic states at the ‘defective’ edge. Comprehensive scanning probe microscopy and spectroscopy, combined with first-principles density functional theory (DFT) calculations, confirmed the successful fabrication of JGNRs possessing ferromagnetically aligned spins localized along the pristine zigzag edge.

Notably, introducing topological antidots within nanographenes provides another powerful means of tailoring their OS multiradical character [57,72]. By selectively removing a *π*-conjugated segment to create an internal cavity or ‘antidot’, the magnetic behavior of the graphene nanostructures can be modulated by the antidot topology [57]. In particular, beyond simply tuning the molecular size, topological engineering via antidot creation in large triangulene homologues enables the fabrication of triangulene quantum rings (TQRs) with precisely tailored spin quantum numbers and magnetic ordering across both the inner and outer zigzag edges (Fig. 3j). Compared with pristine triangulenes, TQRs possess a higher density of zigzag edges per unit area, leading to enhanced spin polarization. Their spin-polarized energy gaps and spin-density distributions can be systematically tuned by adjusting the topology and dimensions of the antidot [73–75]. Antidot engineering has thus emerged as a versatile strategy for constructing novel MGNs with controllable spin states. Recently, we demonstrated a synthetic route employing substituted kekulene as a precursor for the synthesis of unsubstituted [7]TQR featuring a coronene-like central antidot on Au(111) (Fig. 3j) [76]. BR–STM imaging revealed the submolecular structure of [7]TQR, while d*I*/d*V* measurements identified spin-polarized electronic states originating from both its inner and outer zigzag edges. Experimental results, supported by theoretical calculations, confirmed that [7]TQR retains an OS septuple (*S* = 3) ground state on Au(111). This successful demonstration establishes antidot engineering as a promising route for realizing high-spin quantum nanostructures, advancing the design of MGNs.

Beyond these factors, structural parity, particularly in odd-membered systems, introduces geometry-induced spin frustration that further shapes magnetic interaction in *π*-conjugated nanostructures. In such frustrated spin systems, competing magnetic interactions suppress conventional long-range ordering, giving rise to emergent quantum phases and exotic many-body phenomena. Low-dimensional, cyclic and highly symmetric molecular frameworks without edge termination offer ideal platforms for exploring such unconventional spin states [5]. Kawai and colleagues reported *S* = 1/2 antiferromagnetic Heisenberg cyclic pentamer and hexamer formed through the homocoupling of air-stable phenalenyl derivatives [77]. Both exhibit strong magnetic exchange interactions, yet the odd-membered pentamer displays pronounced geometric frustration, which induces rotational symmetry in the spin wave function and results in a 4-fold-degenerate ground state. Spin Hamiltonian calculations further reveal highly degenerated ground states in other odd-membered rings (3, 5 and 7). In parallel, Pavel and co-workers synthesized a cyclopenta-ring-fused oligo(*m*-pheneylene) macrocycle hosting up to nine unpaired *π*-electrons generated via tip-induced dehydrogenation (Fig. 3k) [78]. This system represents the first ‘super radical’ based on the unique *π*-conjugated macrocycle with an odd number of *π*-electrons. Upon adsorption, the macrocycle undergoes a surface-induced distortion to a *D*_3*h*_ symmetry, stabilizing a fully delocalized doublet ground state characterized by a strong delocalized SOMO and global aromaticity.

Collectively, these studies underscore the fundamental influence of molecular topology on the magnetic landscape of graphene nanostructures. By harnessing these topological degrees of freedom, researchers can engineer customized spin configurations and quantum magnetic states, laying the groundwork for magnetic molecular quantum materials and next-generation quantum devices.

### Introducing structural defects

Beyond molecular topology, heteroatom doping and the incorporation of topological defects offer alternative routes to further engineer the magnetic properties of graphene nanostructures [79]. In benzenoid polycyclic hydrocarbons, spin states are primarily determined by their molecular geometries. As stated previously, nanographene flakes must be sufficiently large to host multiple spin centers and prevent hybridization between edge-localized spin states [15–17]. This size requirement restricts the structural diversity of MGNs, as the modulation of spin interactions is confined to geometrical variations within a limited design space. To overcome this constraint, structural defects can be introduced to break the bipartite lattice and half-filling characteristics of ideal benzenoid systems, thereby expanding the accessible magnetic configurations in MGNs.

**Figure 4.**
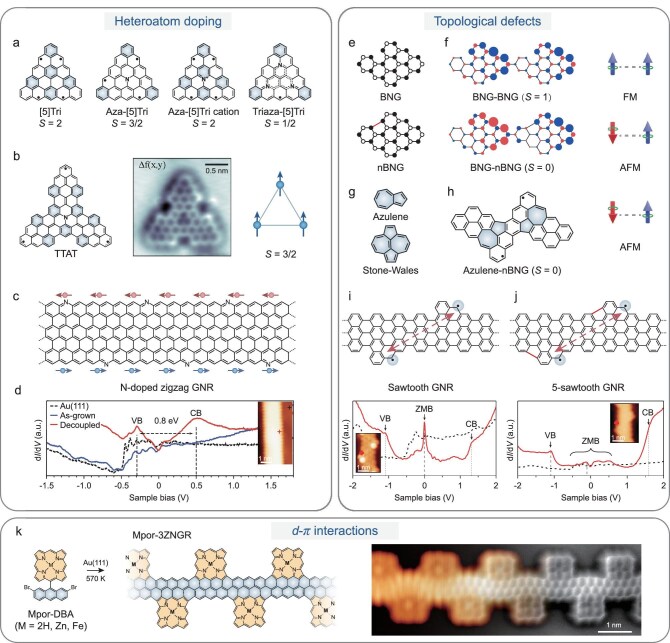
Tuning spin states via introducing structural defects. Defect engineering of spin states and spin interactions in MGNs. (a) Heteroatom doping in triangulene systems and the corresponding spin configurations of pristine [5]triangulene ([5]tri), aza-[5]triangulene (Aza-[5]tri), cationic aza-[5]triangulene and triaza-[5]triangulene (Triaza-[5]tri). (b) Chemical structure of a molecular spin trimer TTAT, its corresponding nc-AFM image and a schematic illustration of the neutral ferromagnetic *S* = 3/2 ground state. Reproduced with permission from Ref. [86]. (c) Chemical structure of a nitrogen-doped ZGNR. (d) d*I*/d*V* point spectra of as-grown and decoupled nitrogen-doped ZGNR on Au(111), along with a reference spectrum from bare Au(111). Reproduced with permission from Ref. [89]. (e, f) Incorporation of a five-membered ring converts bipartite nanographene into non-bipartite nanographene, reversing the interfacial spin density and switching the exchange coupling from ferromagnetic to antiferromagnetic. Reproduced with permission from Ref. [95]. (g) Chemical structures of defects containing multiple non-hexagonal rings: azulene and Stone–Wales defects. (h) Chemical structure of a nanographene incorporating two azulene units. The azulene-type defect breaks bipartite symmetry and stabilizes spin polarization, enabling fine-tuning of magnetic coupling in extended frameworks. (i) Chemical structure of a sawtooth GNR and its corresponding STS spectra, suggesting that periodic sublattice imbalance generates zero-mode bands (ZMBs) confined to one sublattice; (j) chemical structure of a five-sawtooth GNR and its corresponding STS spectra, suggesting that incorporation of five-membered rings mixes sublattice polarization, broadens the ZMB and yields a robust metallic state. Reproduced with permission from Ref. [98]. (k) OSS of MPor-3ZGNR from the precursor (left) and high-resolution STM image gradually overlaid with nc-AFM image of FePor-3ZGNR (right). Reproduced with permission from Ref. [100].

Structural defects can be introduced through either heteroatom doping or the incorporation of topological defects. In heteroatom doping, dopant atoms such as nitrogen (N) or boron (B) are substituted for specific carbon sites and conjugate with the surrounding *p*_z_ orbitals. This substitution influences spin behavior primarily by modifying the electron count and local electronic symmetry. For instance, dopants can inject additional electrons or holes into the *π*-system, decoupling the *π*-electron number from the count of *sp*² carbon sites [80–82]. For instance, [5]triangulene exhibits a ferromagnetic ground state with *S* = 2, whereas substitution with a single nitrogen atom to form aza-[5]triangulene (Fig. 4a) alters the ground state to *S* = 3/2. This change arises from a modified sublattice imbalance and the introduction of lone-pair electrons [83]. Charge transfer also plays a crucial role with the existence of heteroatoms: upon interaction with the Au(111) substrate, cationic aza-[5]triangulene undergoes a Jahn–Teller distortion that restores its spin state to *S* = 2. Increasing the dopant concentration, as in triaza-[5]triangulene [84], further amplifies sublattice imbalance and introduces coupled spin and orbital degeneracy that drive Jahn–Teller distortion and valley mixing in the neutral state, while surface charge transfer stabilizes an OS singlet configuration. In boron-doped systems, such as boron-doped armchair graphene nanoribbon [85], boron substitution interrupts *π*-conjugation and induces two spin-polarized topological boundary states. However, this intrinsic spin polarization becomes spectroscopically observable only when the ribbon is decoupled from the Au(111) substrate, as strong B–Au hybridization otherwise suppresses magnetism. Heteroatom doping has also been extended to larger triangulene-based architectures. By combining pristine and N-doped triangulenes, Vegliante *et al*. have recently synthesized a complex nanographene, tris-triangulene-aza-triangulene (TTAT), incorporating three localized radical units coupled ferromagnetically to yield an *S* = 3/2 ground state (Fig. 4b) [86]. Beyond altering the sublattice topology, heteroatom substitution can also enhance the overall aromaticity of nanographenes. In TTAT, for example, nitrogen incorporation increases the number of Clar’s sextets, strengthening aromatic stabilization and preserving a neutral *S* = 3/2 charge state on the Au(111) surface by suppressing charge transfer.

Moreover, dopants modulate interfacial coupling between graphene nanostructures and metallic substrates. Although ZGNRs are theoretically predicted to host spin-polarized edge states [87,88], early STM studies failed to directly detect such polarization on Au(111) surfaces because of strong edge–substrate hybridization [14]. Blackwell *et al.* overcame this by periodically introducing nitrogen atoms along ZGNR edges (Fig. 4c), which electronically decoupled the edge states from the substrate (Fig. 4d) while stabilizing the zigzag configuration [89]. The nitrogen lone-pair orbitals exhibited strong spin splitting induced by the exchange field of polarized edge carbons, serving as direct spectroscopic signatures of edge magnetism. Thus, heteroatom doping must be carefully designed with respect to both *π*-electron modulation and interfacial charge transfer, as these factors jointly determine the magnetic ground state. Advanced approaches such as N–B co-doping [90] and functionalization with auxiliary substituents [91] further broaden the design space for MGNs.

Topological defects, in contrast, are typically introduced by embedding odd-membered rings (e.g. pentagons or heptagons) into the benzenoid lattice [92–94]. These motifs break the bipartite symmetry of the pristine graphene framework and often lead to unpaired *π*-electrons, thereby enabling tunable spin states. For instance, Zheng *et al.* demonstrated that introducing a five-membered ring into diradical nanographenes reverses the sign of the spin exchange [95,96]. As shown in Fig. 4e and f, a bipartite nanographene unit exhibits an *S* = 1/2 ground state and its dimer displays ferromagnetic coupling. When a pentagon is incorporated into one unit to form a non-bipartite nanographene, the spin density at the interface reverses sign, forcing the exchange interaction to switch from ferromagnetic to antiferromagnetic in order to minimize the on-site Coulomb repulsion. Multiple odd-membered rings, such as azulene units and Stone–Wales defect structures (Fig. 4g), can be simultaneously introduced into graphene nanostructures, further breaking the bipartite symmetry and redistributing the spin density across the entire framework.

As shown in Fig. 4h, structural distortion induced by odd-membered rings enhances exchange interactions between localized spins and allows fine control over magnetic coupling in extended nanographenes [97], which provides an effective strategy to modulate and stabilize diverse spin configurations. A similar topological design that applies to 1D sawtooth graphene nanoribbons can reshape the band structure [98]. As shown in Fig. 4i, their zero-mode bands (ZMBs) arise from periodic sublattice imbalance that produces localized zero-energy spin states on a single sublattice. Introducing five-membered rings along the ribbon edge breaks the lattice symmetry (Fig. 4j), leading to sublattice mixing. The enhanced overlap between neighboring zero-energy modes yields ZMBs with a much larger bandwidth, suggesting a more robust metallic state.

While heteroatom doping and topological defects of non-hexagonal rings mainly regulate magnetism through modifying *π*-conjugation and electron delocalization, an additional control dimension arises from *d–π* interactions. Coupling localized *d*-orbitals of transition metals with delocalized *π* states enables the emergence of hybrid magnetic behaviors. OS metalloporphyrins, for instance, exhibit tunable exchange coupling and spin anisotropy through controlled *d*–*π* hybridization between *π*-radical edges and metal-centered *d* electrons [99]. Extending this concept, Xiang *et al.* integrated iron (Fe) centers into zigzag edges of a graphene nanoribbon (Fig. 4k), where strong d–π coupling mediated by the conjugated backbones produced long-range magnetic ordering [100]. These findings establish *d*–*π* coupling as a versatile mechanism for engineering spin polarization and exchange interactions in MGNs.

By introducing structural defects, one gains multiple avenues to modulate the spin configurations and magnetic interactions in MGNs. However, once the half-filling condition and bipartite symmetry are broken, such systems fall beyond the scope of conventional theoretical frameworks developed for benzenoid nanographenes [8–10]. Moreover, *d*–*π* interactions between transition-metal *d*-orbitals and conjugated *π*-networks introduce additional variables such as magnetic anisotropy, spin–orbit coupling and orbital degeneracy that further complicate the magnetic behavior [99,100]. While empirical observations and computational studies have provided valuable insights into these complex spin systems, a more comprehensive theoretical framework is still required to accurately describe non-benzenoid and *d*–*π*-coupled systems and to predict their structure–property correlations and magnetic exchange mechanisms.

## POLYRADICAL NANOGRAPHENES WITH STRONG SPIN ENTANGLEMENT

Spin-correlated systems, hosted in low-dimensional materials, offer an exciting platform for exploring exotic quantum phases such as quantum spin liquids [101]. Recent advances in electron spin resonance STM (ESR–STM) have demonstrated that singlet–triplet transitions in atomic spin systems can be engineered to form decoherence-free subspaces that are intrinsically resistant to magnetic fluctuations and well suited for encoding quantum information [102,103]. Owing to their long spin coherence times, stemming from negligible hyperfine interaction and spin–orbit coupling [66], carbon-based radicals, particularly correlated polyradical nanographenes, hold significant promise as robust molecular qubits for constructing scalable and complex quantum networks [104]. However, realizing these architectures remains challenging, as polyradical nanographenes exhibit high intrinsic reactivity and poor solubility, imposing constraints on both solution-phase synthesis and OSS.

**Figure 5.**
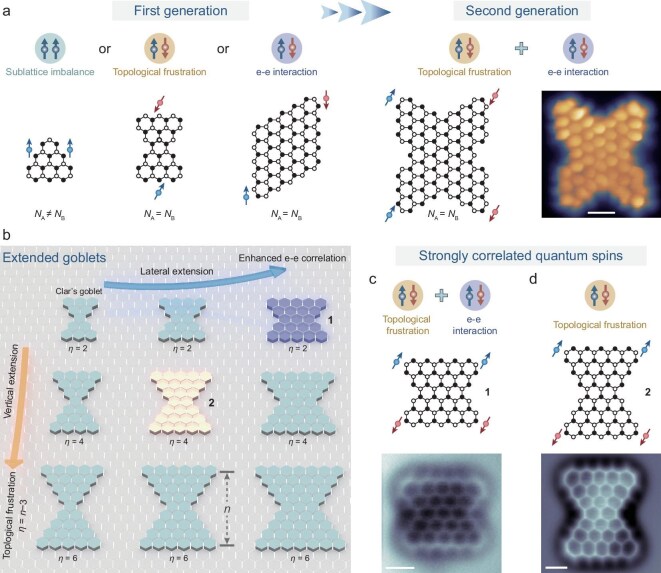
Polyradical nanographenes with strong spin entanglement. (a) Left: Schematic illustration of representative first-generation OS nanographenes (triangulene, Clar’s goblet and rhombene) with different single *π*-magnetism origins, including topological frustration (sublattice imbalance) and e–e interactions. Right: Schematic illustration of a representative second-generation OS butterfly-shaped nanographene with multiple *π*-magnetism origins, along with its corresponding BR–STM image. Reproduced with permission from Ref. [13]. (b) Illustration of OS nanographenes with varying sizes obtained by lateral and vertical extensions of Clar’s goblet. Here, *η* denotes the number of ZMs in a nanographene and *n* denotes the total number of benzene-ring rows. (c) *π*-magnetism origin and chemical structure of molecule **1** (top), together with its corresponding nc-AFM image (bottom). (d) *π*-magnetism origin and chemical structure of molecule **2** (top), together with its corresponding nc-AFM image (bottom). Reproduced with permission from Ref. [105]. *N*_A_ and *N*_B_ denote the number of sublattices A and B. All scale bars: 0.5 nm.

As previously discussed, OS nanographenes can exhibit unconventional *π*-magnetism originating from sublattice imbalance, topological frustration or strong e–e interactions (Fig. 5a). Conventional molecular design strategies, however, typically focus on exploiting only one of these mechanisms at a time, thus limiting both the number of correlated spins and the diversity of accessible magnetic orderings. Recently, our group reported the first example of a fully fused butterfly-shaped tetraradical nanographene on Au(111), which simultaneously integrates both topological frustration and e–e interactions (Fig. 5a, right panel) [13]. BR–STM imaging reveals that its molecular geometry imparts a topologically frustrated network with a nullity of two (*η* = 2), implying the presence of two ZMs. Meanwhile, a small energy gap triggers the spin-symmetry breaking driven by e–e interactions, contributing two additional radicals. The resulting four spins interact through a combination of ferromagnetic and antiferromagnetic couplings, resulting in a many-body singlet ground state with pronounced spin entanglement. STS measurements, coupled with many-body calculations, confirm the tetraradical character, exhibiting a singlet–triplet excitation energy of 9 meV. Further insights into the spin states and correlated spin behaviors were obtained by using a nickelocene-functionalized magnetic tip.

Advancing both the fundamental understanding of MGNs and their potential in future quantum technologies demands the development of a broader library of entangled polyradical nanographenes with tunable spin numbers and coupling strengths. Toward this goal, our group recently introduced a predictive framework based on a Clar’s goblet extension principle [105], enabling the rational design of correlated polyradical nanographenes with tailored spin numbers and enhanced resilience to external perturbations. As shown in Fig. 5b, this framework identifies two independent mechanisms for radical generation. Vertical structural extension of the Clar’s goblet motif increases the number of topologically frustrated ZMs, which scale linearly with the total number of benzene rows (*n*). In contrast, lateral extension enhances orbital delocalization and strengthens e–e interactions, thereby yielding additional spin states. By tailoring these structural motifs, we synthesized two Clar’s goblet homologues, C_62_H_22_ (**1**) and C_76_H_26_ (**2**), via OSS on Au(111). High-resolution nc-AFM images (Fig. 5c and d, bottom panels) confirm the successful fabrication of both nanostructures, while STS measurements complemented by multireference theoretical calculations reveal their correlated tetraradical ground state with strong spin entanglement in each case. Notably, the tetraradical nature of C_76_H_26_ (**2**) arises solely from topological frustration associated with vertical extension, whereas that of C_62_H_22_ (**1**) results from synergistic contributions from both the topological frustration and e–e interactions linked to lateral extension. Both systems feature many-body singlet ground states, with measured excitation energies of 30 meV (**1**) and 9 meV (**2**), respectively. These results establish, for the first time, experimentally and theoretically validated structure–property relationships within the Clar’s goblet family, laying the groundwork for designing scalable polyradical nanographenes with predictable correlated spin behaviors.

The fabrication of polyradical nanographene has long been regarded as formidable, largely because preparing extended, fully conjugated frameworks becomes increasingly challenging as the molecular size grows. Beyond intramolecular cyclization strategies, such as those employed in butterfly-shaped nanographene and extended Clar’s goblets, bottom-up approaches that assemble smaller, rationally designed precursors through intermolecular coupling offer expanded design flexibility. This strategy is particularly advantageous for constructing extended multi-spin systems, the high molecular weight of which often limits precursor evaporability and stability. As shown in Fig. 3d, the spin coronoid is constructed from six molecular precursors, where the bromo substituents promote intermolecular coupling and macrocycle formation on the Au(111) surface [60]. Despite these advances, achieving a deeper understanding of strongly entangled polyradical nanographene will require further synthetic innovation as well as theoretical frameworks capable of describing many-body spin phases.

## ADVANCES IN TECHNIQUES FOR PROBING NANOSCALE MAGNETISM

The successful experimental realization of nanographenes with strongly correlated spin states underscores the urgent need for advanced characterization tools capable of resolving their complex magnetic nature. While conventional STM and STS have been instrumental in visualizing chemical structures and probing local electronic states at the single-molecule level, they lack direct sensitivity to spin orientation and magnetic coupling. This limitation renders them insufficient for capturing the subtle spin ordering, multi-spin entanglement or low-energy magnetic excitations that are central to MGNs. To overcome these limitations and gain a deeper insight into nanoscale magnetism, it is essential to further develop novel spin- and time-resolved probing techniques. The following section will introduce recent advances in magnetism-probing methods that extend beyond the capabilities of conventional scanning probe techniques, enabling detailed characterization of the spin phenomena in molecular quantum materials.

While the presence of edge states in ZGNRs [14] and chiral GNRs (chGNRs) [106] has been confirmed experimentally, obtaining direct evidence of their spin polarization, which is a central question of carbon-based magnetism, remains challenging. In this context, spin-polarized STM (SP–STM) provides a unique approach to probing spin-dependent electronic states with atomic resolution [107]. By employing a magnetic tip, either fabricated from a bulk magnet or realized by using ferromagnetic coatings such as Fe, Co or Cr, SP–STM enables spin-dependent tunneling, thereby providing access to local magnetic contrast beyond conventional STM. However, the intrinsically weak spin–orbit coupling of *sp*^2^ carbon, often regarded as a key advantage of *π*-magnetism for achieving long spin coherence times, simultaneously imposes the main limitation: the near absence of magnetic anisotropy, which hampers the detection of stationary spin moments. Recently, Brede *et al*. reported the detection of spin-polarized edge states in chGNRs using SP–STM by synthesizing the ribbons on a ferromagnetic GdAu_2_ monolayer [108]. The substrate-induced exchange interaction effectively stabilizes the organic magnetic moments against thermal and quantum fluctuations. This strategy allows the extraction of the energy-dependent spatial distribution of spin polarization associated with extended *π*-orbital edge states, going beyond the detection of localized magnetic moments typically confined to radical sites.

**Figure 6.**
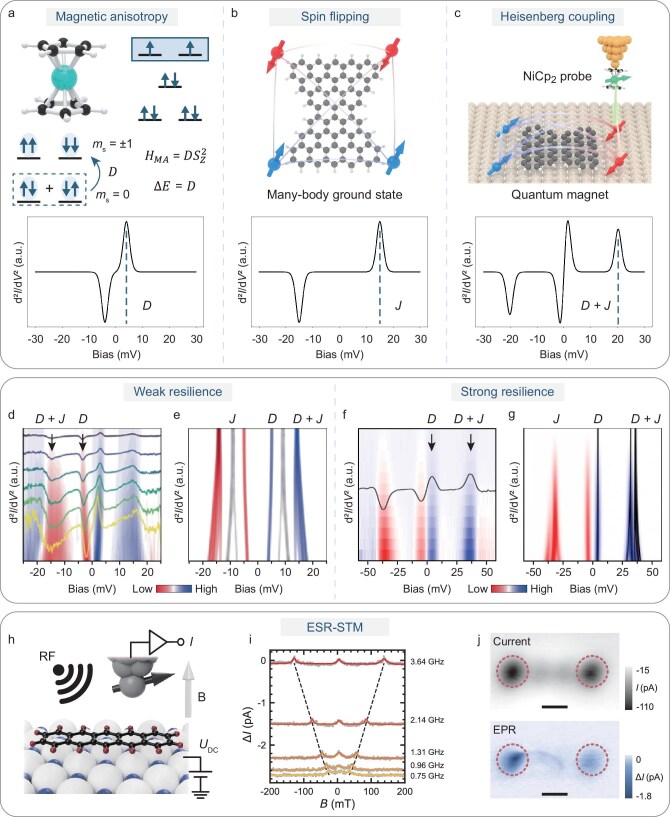
Advances in techniques for probing magnetism. (a) Chemical structure of a NiCp_2_ and schematic diagram of its spin state energy levels (top) and calculated IETS spectrum of easy-plane magnetic anisotropy excitation of NiCp_2_ (bottom). (b) Chemical structure of molecule **2** (top) and calculated IETS spectrum of its spin flipping excitation (bottom). (c) Schematic illustration of the measurement setup of molecules with a NiCp_2_ tip (top) and calculated IETS spectrum of coupled spin excitations between a NiCp_2_ tip and the measured molecule (bottom). (d) IETS spectra plotted in a color scale taken over the corner of butterfly-shaped nanographene (Fig. 5a) overlaid with the corresponding IETS spectra taken at different tip–sample distances. Arrows indicate the energies of NiCp_2_ MA excitation and coupled spin excitation, respectively. (e) Calculated IETS spectra plot of butterfly-shaped nanographene as a function of the coupling strength *J*. Reproduced with permission from Ref. [13]. (f) IETS spectra plotted in a color scale taken over the corner of molecule **1** (Fig. 5c) taken at different tip–sample distances, overlaid with one representative IETS spectrum. Arrows indicate the energies of NiCp_2_ MA excitation and coupled spin excitation, respectively. (g) Calculated IETS spectra plot of molecule **1** as a function of the coupling strength *J*. Reproduced with permission from Ref. [105]. (h) Sketch of the ESR–STM junction. The magnetic tip is shown on top of a pentacene molecule on MgO/Ag(100). (i) Electron paramagnetic resonance (EPR) spectra as a function of *B* acquired at different frequencies. The dashed lines show the shift of the resonance field *B*_0_ with frequency. (j) Current map (top) taken at constant height and simultaneous map (bottom) of the EPR signal. Reproduced with permission from Ref. [117]. All scale bars: 0.5 nm.

Another powerful approach uses STM tips functionalized with a magnetic molecule as a spin sensor, allowing the direct detection of magnetic excitations corresponding to transitions between discrete energy levels. When such a functionalized tip is positioned above specific sites on a sample, local perturbations to the energy levels of the sensor, manifesting as shifts or splittings in the spectra, provide valuable information about local magnetic interactions, including Coulomb interactions, Heisenberg exchange and dipolar couplings. Nickelocene (NiCp₂)—a metallocene with a triplet ground state (*S* = 1)—has emerged as a powerful spin sensor for this purpose (Fig. 6a). NiCp₂ features a well-defined magnetic anisotropy (MA) excitation between the in-plane (*m*_s_ = 0) and out-of-plane (*m*_s_ = ±1) spin states, typically observed as a symmetric peak–dip feature in the second derivative (d²*I*/d²*V*) spectra near an energy of ∼3–5 meV (Fig. 6a, bottom) [109]. Perturbations from nearby magnetic moments cause this MA excitation to shift or split due to additional dipole–dipole, Zeeman or exchange interactions, enabling NiCp₂ to act as a highly sensitive and spatially precise probe of the local magnetic environment [110].

When positioned above a molecule magnet (e.g. butterfly-shaped nanographene and extended Clar’s goblets), the NiCp_2_-functionalized tip senses both its own MA excitation (*D* ∼5 meV, Fig. 6a) and additional molecular spin excitations (e.g. internal exchange energy *J* ∼15 meV, Fig. 6b). The emergence of new features at energies corresponding to sums of *J* and *D* (Fig. 6c) indicates coupled spin excitations. As shown in Fig. 6d and f, experimentally, the *J* step may appear weak or even absent due to the dominant inelastic channel associated with the NiCp_2_ excitation [109]. As the tip approaches the molecule by decreasing the tip–sample distance, the Heisenberg exchange interaction (*J*_int_) between the NiCp₂ spin and the molecular spin strengthens. This increased coupling leads to mixing between nominal single- and double-spin excitations, resulting in nonlinear shifts and splittings in the excitation spectrum. By analysing these features, along with their distance-dependent *E*–*J* evolution (Fig. 6d–g), one can extract key physical parameters such as molecular-level spacings, spin configuration and gap inversions.

Pioneering work by Wilson and colleagues used STM to characterize isolated nickelocene and nickelocene pairs through Heisenberg interaction simulation [111]. Limot and co-workers then demonstrated nickelocene as a spin sensor for measuring the spin polarization of magnetic adatoms [110]. Building on these foundations, Song *et al.* employed a NiCp₂-functionalized tip to probe a butterfly-shaped tetraradical nanographene, observing site-specific spin flips and exchange coupling with submolecular precision [13]. The attachment of NiCp₂ to the STM tip suppressed the original molecular excitation (∼9 meV) and introduced a prominent MA excitation peak, alongside an additional double-excitation feature involving both NiCp₂ and the molecular spin (Fig. 6d). By tracking the shifts and splittings of the MA peak, the tetraradical spin configuration was resolved in good agreement with many-body calculations. More recently, Urgel and colleagues further expanded single-molecule magnetic characterization by demonstrating that an STM equipped with a NiCp₂-functionalized probe can discriminate between nearly degenerate multireference ground states in individual MGNs and map the spatial distribution of their exchange interactions [112].

Furthermore, our group extended this technique to probe magnetic resilience in polyradical nanographenes, thereby establishing a robust protocol for extracting intramolecular interaction parameters [105]. Using NiCp₂-functionalized scanning probe techniques, we experimentally validated the molecule-dependent resilience of their correlated singlet ground states, demonstrating the stability and generality of this methodology across distinct molecular systems. As shown in Fig. 6f, distance-dependent measurements on molecule **1** exhibit only minor energy shifts upon decreasing distance (increasing *J*_int_), in stark contrast to the larger shifts observed for the butterfly-shaped nanographene (Fig. 6d) and molecule **2**. These differences reflect varying degrees of spin resilience and highlight the potential of polyradical nanographenes to function as molecular-scale spin sensors or robust qubits.

Complementary to IETS-based approaches, which are limited by thermal broadening, electronic spin resonance combined with STM (ESR–STM) offers unprecedented energy resolution down to tens of nano-electron volts and enables coherent control of individual spins on the surface [113]. In an ESR–STM setup (Fig. 6h), a radio-frequency (RF) bias is applied across the tunneling junction and drives spin resonance when the RF frequency matches the Larmor frequency of an adsorbed spin center. During ESR measurements, a spin-polarized tip assists the driving and probes the measurable change in the tunneling current induced by the spin transition (Fig. 6i). This technique has facilitated groundbreaking studies of probing and engineering quantum spin systems, including the construction of clock transitions in a strongly coupled spin system, as mentioned above.

Beyond atomic spins, Zhang *et al.* and Kawaguchi *et al.* resolved single-molecule ESR signals arising from *d*-orbitals of metal centers and *π*-radical spin, respectively, enabling precise determination of the *g*-factor and MA [114,115]. Moreover, by employing resonant RF pulses, Willke *et al.* demonstrated Rabi oscillations and Hahn-echo measurements of single molecules [116]. Besides, Kovarik *et al.* utilized ESR–STM to inject spin-polarized currents into a pentacene molecule, achieving dynamic control of single spins with a spin–orbit torque effect (Fig. 6h–j) [117]. More recently, Esat *et al*. demonstrated a quantum sensor on the tip that can be addressed by using ESR, paving the way for mapping electrical and magnetic field variations within a nanostructure [118]. These advances firmly position ESR as a powerful complement to the conventional STM toolbox, unlocking access to coherent quantum control and single-molecule magnetism with unprecedented precision.

Other advanced techniques include combining an optical setup with STM, such as STM-induced luminescence (STML) and terahertz STM (THz–STM), which extend measurements beyond the spin degree of freedom. STML relies on an incident laser beam or tunneling electrons to excite the electronic states of a nanostructure, the luminescence of which is significantly enhanced by the nanocavity of the tunneling junction. Detecting the resulting photon emission enables simultaneous sub-nanometer spatial resolution and micro-electron volt energy resolution [119], allowing direct access to molecular excited states, plasmonic modes, charge-transfer processes and radiative transitions at the single-molecule level [120]. Recently, Schull and co-workers achieved atomically resolved fluorescence spectroscopy, revealing a sharp emission from a long-lived dark exciton localized at the topological ends of graphene nanoribbons [121]. These topological end states host unpaired electrons and are therefore spin-polarized, offering versatile organic quantum architectures that combine electronic, magnetic and photonic degrees of freedom. THz–STM is an ultrafast STM technique in which terahertz (THz) pulses are coupled into the STM junction to drive electron tunneling on femtosecond timescales [122]. The THz field transiently modulates the tunneling barrier, enabling the measurement and control of ultrafast measurements of wave-function dynamics at the atomic scale [123]. Using this approach, Huber and co-workers have probed the femtosecond-scale quantum motion of molecules on the scale of a single electronic orbital, ranging from vibrations to electronic excitations [124]. These optical approaches further expand the capabilities of graphene nanostructures, highlighting their potential as a versatile platform for spin-photon/phonon interfaces as well as quantum emissions.

Together, these emerging experimental methodologies, spanning functionalized IETS probes and STM combined with electromagnetic waves, extend the capabilities of conventional scanning probe techniques, enabling the direct visualization, manipulation and characterization of magnetic interactions at the atomic scale. They open up new frontiers in molecular magnetism and quantum information science, and provide powerful tools for engineering designer spin systems in molecular quantum materials.

## CONCLUSIONS AND PERSPECTIVES

Over the past decades, the rapid development of OSS and advanced scanning probe microscopy has enabled the atomically precise fabrication and characterization of MGNs exhibiting unconventional magnetism and quantum properties [11–13,17]. The establishment of rigorous structure–spin correlations across diverse graphene nanostructures represents a critical step toward predictable spin engineering in MGNs. Moreover, rational molecular topology design and controlled defect incorporation have been effectively employed to tailor electronic configurations, magnetic ordering, spin multiplicity and exchange coupling, pushing the field toward tunable organic magnets and carbon-based spintronic components [1,2].

Building on these advances, the exploration of polyradical nanographenes featuring strong spin entanglement has opened up a new paradigm for MGNs, demonstrating how *π*-conjugated frameworks can host correlated spin networks with tunable coupling strengths [5,6,13,77]. In particular, 1D covalently bonded OS nanographenes, formed by linking individual units into extended architectures, exhibit robust and tunable magnetic coupling between *π* spins [125–128]. These systems provide a versatile platform for studying highly entangled quantum spin states and represent potential candidates for spintronic applications. Recent progress in molecular precursor design, combined with bottom-up OSS, has enabled the fabrication of such chains with atomic precision, offering unprecedented control over their structure and magnetic properties [5,129]. A prominent example is the antiferromagnetic *S* = 1 spin chain, long predicted to feature a Haldane gap, strong quantum fluctuations and fractionalized *S* = 1/2 edge states protected by topological symmetry [130]. These hallmark features have now been experimentally confirmed in chains constructed from [3]triangulene units [5] and metal-free porphyrins [129]. STS revealed gapped bulk excitations and localized fractional edge states, in agreement with Heisenberg model predictions. In addition, antiferromagnetic *S* = 1/2 chains further broaden the accessible landscape of quantum magnetism. Alternating-exchange chains support symmetry-protected topological phases with termination- and parity-dependent edge degeneracies [131–133], while uniform Heisenberg chains realize gapless spin-liquid ground states dominated by quantum fluctuations [6]. Recent bottom-up and pre-protected synthetic strategies have enabled the realization of long, well-controlled chains, revealing odd–even effects, vanishing excitation gaps in the thermodynamic limit [6] and fractional spinon excitations the dispersion of which can be directly mapped [134,135]. Together, these developments establish nanographene-based spin chains as powerful and highly tunable model systems for investigating correlated quantum magnetism and topological many-body physics.

Nevertheless, the realization of such spin systems in a scalable, highly selective and stable manner remains a long-standing challenge in OSS and demands further advances. Side reactions are often unavoidable, especially at elevated annealing temperatures or in the presence of highly reactive intermediates and products. One effective approach to improving reaction selectivity is to lower the activation barriers. For instance, the deposition of metal atoms [136] and the dosing of atomic hydrogen [137] have been shown to catalyse Ullmann coupling and cyclodehydrogenation reactions, thereby enhancing both selectivity and yield. Such catalytic approaches provide promising pathways toward scalable OSS. Atomic manipulation offers an alternative strategy by enabling precise single-molecule reactions that are difficult to realize through purely thermo-assisted processes [19]. However, in contrast to thermal activation, atomic manipulation typically suffers from low production efficiency. In this context, integrating scanning probe techniques with artificial intelligence (AI) has emerged as a promising route to overcoming this limitation [27]. Recent advances demonstrate that AI, particularly reinforcement learning, can fundamentally transform OSS and atomic-scale manipulation by enabling autonomous, data-efficient control in scanning probe experiments [138,139]. Deep and reinforcement learning algorithms have been shown to learn optimal manipulation strategies for atoms and single molecules, overcoming unknown manipulation parameters, tip instabilities and complex tip–adsorbate interactions [138,139]. Beyond individual manipulation tasks, integrated AI frameworks combining deep reinforcement learning, Bayesian optimization and DFT enable autonomous on-surface chemical reactions and molecular construction [140]. Together, these approaches establish AI-driven scanning probe microscopy as a powerful paradigm for the scalable, adaptive and insight-generating fabrication of atomically precise nanostructures.

Complementing synthetic progress, the rapid evolution of experimental magnetometry has opened up unprecedented opportunities for probing spin phenomena with atomic precision. Techniques such as inelastic IETS, SP–STM and ESR–STM now enable the resolution of magnetic excitations, spin anisotropies and coherent transitions within individual molecules. Integrating these approaches with ultrafast optical probes and spin manipulation techniques promises access to dynamic spin phenomena, non-equilibrium magnetic switching and decoherence pathways with unmatched precision. These multimodal approaches are essential for linking microscopic magnetism to macroscopic behavior, and ultimately to device performance.

Despite these remarkable advances, several challenges remain before MGNs can evolve from model systems into functional quantum devices [141,142]. Theoretically, developing unified frameworks capable of accurately describing non-bipartite and correlated spin systems remains a key frontier. Bridging this gap will require integrating graph theory with multireference quantum chemistry, Hubbard-type models and machine-learning-based predictive tools. Experimentally, preserving magnetism under ambient or device-relevant conditions remains nontrivial, as substrate interactions, charge transfer and spin–orbit coupling can suppress magnetic behavior or alter exchange pathways. Decoupling strategies using insulating interlayers such as NaCl and boron nitride will be essential for achieving coherent spin control and readout using ESR–STM [113], as well as for enabling efficient photon emission [120,121]. Moreover, while the transfer protocols developed for large-sized graphene flakes have been adapted to bottom-up fabricated nanostructures [143], extending these methods to chemically reactive MGNs remains elusive. Inspired by recent demonstrations of armchair-edged nanographenes and polyanthrylene synthesized on TiO_2_ [144,145], direct synthesis on insulating or semiconducting substrates may offer a promising route towards device-compatible architectures. For spintronic applications, the formation of well-defined interfaces, commonly referred to as spinterface, between MGNs and ferromagnetic substrates is a critical requirement. In this context, rare-Earth surface alloys such as GdAu_2_ [146] and TbAu_2_ [147] have emerged as particularly attractive platforms, as they exhibit stable magnetic order at low temperatures and can effectively interact with the magnetism of molecular adsorbates. Notably, these surface alloys can efficiently catalyse Ullmann-type polymerization reactions, enabling the bottom-up fabrication of MGNs directly on magnetic substrates [148,149], while exhibiting long-range ferromagnetic order with pronounced easy-plane anisotropy at a liquid-helium temperature. Recent studies report a symmetric Kondo-resonance splitting for [2]triangulene on TbAu_2_ [150], indicating strong proximity-induced exchange interactions, and show that topologically nontrivial chGNRs synthesized on GdAu_2_ either retain a charge-neutral diradical ground state or convert to a singly anionic doublet configuration [151]. Together, these findings establish rare-Earth surface alloys as versatile platforms for preserving and controlling spin states, enabling their integration into future molecular spintronic devices.

Looking ahead, future progress will rely on the synergistic integration of synthetic chemistry, surface science and quantum measurement to realize scalable, defect-tolerant architectures with programmable spin functionalities. By translating fundamental insights into design principles for controllable spin systems, MGNs may transition from theoretical constructs to practical components for quantum spintronics, molecular qubits and correlated quantum simulators [1–7]. The rapid and intertwined progress of theory and experiments continues to inspire the material-science community, driving the discovery of new quantum phenomena and steadily expanding the horizons of molecular quantum materials.
